# Intravenous Drug Abuse by Patients Inside the Hospital: A Cause for Sustained Bacteremia

**DOI:** 10.1155/2016/1738742

**Published:** 2016-06-28

**Authors:** Noopur Goel, Lubna Bashir Munshi, Braghadheeswar Thyagarajan

**Affiliations:** Department of Medicine, Monmouth Medical Center, Long Branch, NJ 07740, USA

## Abstract

Patients with history of intravenous drug abuse are noted to be at risk of several infections including HIV, endocarditis, and other opportunistic infections. We report the case of a patient with sustained* Bacillus cereus* bacteremia despite use of multiple antibiotic regimens during his inpatient stay. Our case highlights the importance of high suspicion for active drug use inside the hospital in such patients. This is important in order to minimize unnecessary diagnostic workup and provide adequate treatment and safe hospital stay for these patients.

## 1. Introduction

Intravenous drug abuse is a major health concern in the United States often leading to many medical conditions. These individuals often go to great extents to procure illegal contraband inside the hospital through third party visitors. This often leads to variation in their clinical condition and misleading false diagnosis by the physicians. We report a case of a 25-year-old Caucasian male initially admitted for* Bacillus cereus* bacteremia later found to be abusing heroin in the hospital leading to a prolonged stay.

## 2. Case Presentation

Our patient was a 25-year-old Caucasian male who presented to the hospital with the chief complaint of fever and night sweats. Onset of symptoms was four weeks prior at which time he presented to another hospital. His initial workup included an X-ray and CT scan of his chest, which showed bilateral consolidation. His blood cultures grew* Flavimonas* for which he was started on appropriate antibiotics. The patient left that hospital against medical advice before the completion of treatment. The patient reported persistent symptoms of fever, chills, and night sweat since then. His past medical and surgical history is significant only for nephrolithiasis status after lithotripsy procedure. As per patient, his social history was significant for smoking about 1 pack of cigarettes daily and he denied any alcohol or drug abuse. His family history was noncontributory and he has no known allergies. The patient also denied any recent travel or sick contacts.

When the patient presented to the emergency room at our institute, his vital signs were the following: blood pressure of 126/54 mm of Hg, heart rate of 102 bpm, respiratory rate of 18/min, and a temperature of 101.2°F. His respiratory system examination revealed clear breath sounds on auscultation and his cardiac examination was unremarkable. His head and neck and neurological and abdominal examination were also unremarkable. However, on generalized skin examination there were signs of injection usage in his right and left antecubital fossa. His initial blood work revealed a white blood cell count of 11700 with 87% neutrophils and 79 bands, a normal hematocrit and platelet count. His renal functions, liver functions, and urinalysis were within normal limits. Blood cultures were redrawn from peripheral sites and grew* Bacillus cereus* which was found to be resistant to penicillin in multiple culture bottles. Chest X-ray was unremarkable.

Even after 9 days of treatment with IV vancomycin, he continued to have positive blood cultures for bacillus cereus. Hence a transesophageal echocardiogram was performed to evaluate for cardiac valves vegetation, which was negative. Ultimately, a urine toxicology screen was performed during this hospital stay which was positive for opioids. On further detailed questioning, he admitted to a history of intravenous heroin drug abuse. Hence, it was determined that his* Bacillus cereus* bacteremia was secondary to IV drug abuse and possibly the patient active drug abuse inside the hospital. The patient was placed on a 24-hour one-to-one close observation along with visitor screening, as his behavior was harmful to himself. He was then transitioned to Daptomycin. Eventually his repeat blood cultures no longer grew* Bacillus cereus* or any other microbe. The patient was counselled regarding the deleterious effects of IV drug abuse. He was eventually discharged to home without an IV line and instructions were given to return to the hospital daily for completion of his antibiotics under nursing supervision.

## 3. Discussion

In 2013, it was estimated that around 4.8 million people in USA have used heroin at some point in their lives and around 289,000 accepted to use it in the last month [1]. The number of deaths due to heroin overdose has increased considerably over the years with about 8257 reported in 2013 [2]. Nonmedical use of opioid analgesics has led to increase in use of heroin [3]. Opioids act on transmembrane neurotransmitter receptors such as mu, kappa, and delta.* *The activation of mu receptors results in the effects of analgesia, euphoria, and withdrawal. They are mediated through the G proteins [4]. Often, heroin is used in the form of intravenous drug, leading to repeated use of needles, which increases the risk of infections. Needle sharing increases the risk of transmission of blood borne pathogens.

*Bacillus cereus* bacteria are often found in their spore form able to survive the harshest of environmental conditions such as contaminated needles [5]. Apart from gastrointestinal illness,* Bacillus cereus* is known to cause other infections involving the musculoskeletal, respiratory, ocular, central nervous systems, and cardiovascular systems in intravenous drug users [6]. There have been reports of* Bacillus cereus* causing infections such as cellulitis, endocarditis, and panophthalmitis [7–10]. With prompt initiation of appropriate antibiotics, the prognosis is often good [11]. High morbidity and mortality are observed in patients with prosthetic valve endocarditis often requiring valve replacement [10]. Dancer et al. reported finding a relation between the* Bacillus cereus* identified in the patient's blood sample and the heroin possessed by the patient at a molecular level [7]. Benusic et al. reported the analysis of the heroin found in their community through the assistance of police; they were able to able grow* Bacillus cereus*, coagulase-negative* staphylococcus aureus*; and* Escherichia vulneris* [12]. This signifies that often* Bacillus cereus* is a contaminant in heroin which leads to a high rate of infection in intravenous drug abusers. In our patient he just had bacteremia with* Bacillus cereus* and* Flavimonas* without systemic complications. Unfortunately, we were not able to acquire the heroin the patient used for the purpose of culture.

Gastrointestinal infection of* Bacillus cereus* does not indicate treatment. Other systemic infections require treatment with intravenous antibiotics ranging from two to four weeks for bacteremia and four to six weeks for endocarditis [11].* Bacillus cereus* produce beta lactamase and are resistant to penicillin, cephalosporins, and trimethoprim/sulfamethoxazole. Reports describe complete susceptibility to vancomycin, quinolones, gentamicin, carbapenems, and tigecycline and intermediate susceptibility to clindamycin, tetracycline, and erythromycin [13]. Our patient did not have any systemic complication, he was initially treated with vancomycin and eventually transitioned to daptomycin for once daily admission regimen and completed four weeks of antibiotic therapy.

Intravenous drug abuse is a public problem in the United States. Often these patients are admitted to the hospital for multiple medical conditions secondary to their usage of intravenous drugs themselves. It is quite difficult for 24-hour monitoring of the patient in their private room during their hospital stay. It is more difficult to restrict visitors to the patient as it is the right of every patient to see their family and friends during their hospital stay. At times, visitors are known to bring patients harmful contraband such as needles and drugs which jeopardizes patient safety and care. Complications from these items mislead the physicians to false differential diagnosis leading to unnecessary diagnostic testing and treatment thereby increasing medical cost. As in our patient, he had access to recreational drugs through a third party, leading to continued bacteremia and exposure, despite multiple antibiotics. As a result, healthcare costs were significantly increased. Furthermore, the patient was continually at risk for more serious complications due to prolonged bacteremia. After implementing 24-hour close observation, we were able to prevent further exposure and treat the patient appropriately. Harmful consequences from potential recreational drug use in a hospital setting are a common issue faced by many clinicians.

## 4. Conclusion

Our case exemplifies the importance of considering inpatient intravenous drug abuse in patients with prolonged bacteremia and known history of intravenous drug abuse. These cases test the physicians' investigative skills with the fine balance of trusting the patient at the same time. We need to find a proper balance between implementation of stricter policies in the hospital and possible security screening of visitors without violating patient rights. Thus improving patient care and reducing overall hospital cost.

## References

[1] (2014). *2013 National Survey on Drug Use and Health: Detailed Tables*.

[2] Jones C. M., Logan J., Gladden R. M., Bohm M. K. (2015). Vital signs: demographic and substance use trends among heroin users—United States, 2002–2013. *Morbidity and Mortality Weekly Report*.

[3] Jones C. M. (2013). Heroin use and heroin use risk behaviors among nonmedical users of prescription opioid pain relievers—United States, 2002–2004 and 2008–2010. *Drug and Alcohol Dependence*.

[4] Camí J., Farré M. (2003). Drug addiction. *The New England Journal of Medicine*.

[5] Weller P. F., Nicholson A., Braslow N. (1979). The spectrum of *Bacillus bacteremias* in heroin addicts. *Archives of Internal Medicine*.

[6] Bottone E. J. (2010). Bacillus cereus, a volatile human pathogen. *Clinical Microbiology Reviews*.

[7] Dancer S. J., McNair D., Finn P., Kolsto A.-B. (2002). Bacillus cereus cellulitis from contaminated heroin. *Journal of Medical Microbiology*.

[8] Shamsuddin D., Tuazon C. U., Levy C., Curtin J. (1982). Bacillus cereus panophthalmitis: source of the organism. *Reviews of Infectious Diseases*.

[9] Craig C. P., Wie Shing Lee, Monto Ho (1974). Bacillus cereus endocarditis in an addict. *Annals of Internal Medicine*.

[10] Thomas B. S., Bankowski M. J., Lau W. K. K. (2012). Native valve *Bacillus cereus* endocarditis in a non-intravenous-drug-abusing patient. *Journal of Clinical Microbiology*.

[11] Bantados B., Grein J., Ewing A. (2013). *B. cereus* bacteremia in an IV drug abusing patient. *Proceedings of UCLA Healthcare*.

[12] Benusic M. A., Press N. M., Hoang L. M., Romney M. G. (2015). A cluster of Bacillus cereus bacteremia cases among injection drug users. *The Canadian Journal of Infectious Diseases & Medical Microbiology*.

[13] Luna V. A., King D. S., Gulledge J., Cannons A. C., Amuso P. T., Cattani J. (2007). Susceptibility of Bacillus anthracis, Bacillus cereus, Bacillus mycoides, Bacillus pseudomycoides and Bacillus thuringiensis to 24 antimicrobials using Sensititre® automated microbroth dilution and Etest® agar gradient diffusion methods. *Journal of Antimicrobial Chemotherapy*.

